# Nasopharyngeal Bacterial–Fungal Dysbiosis in Respiratory-Diseased Endangered Forest Musk Deer (*Moschus berezovskii*)

**DOI:** 10.3390/biology15070587

**Published:** 2026-04-06

**Authors:** Lijuan Suo, Kun Bian, Jie Tang, Feiran Li, Kuo Sun, Chao Yang

**Affiliations:** Shaanxi Key Laboratory of Qinling Ecological Security, Shaanxi Institute of Zoology, Xi’an 710032, China

**Keywords:** forest musk deer (*Moschus berezovskii*), nasopharyngeal microbiome, respiratory disease, microbial dysbiosis, bacterial–fungal community

## Abstract

Background: The nasopharyngeal microbiome is crucial for respiratory health in mammals, yet it remains poorly characterized in the endangered forest musk deer (*Moschus berezovskii*), particularly in the context of disease. Methods: We compared the bacterial (16S rRNA) and fungal (ITS2) communities in the nasopharynx of healthy (*n* = 6) and clinically diseased (*n* = 6) individuals. Results: Although alpha diversity did not differ significantly, beta diversity (PCoA) analysis revealed distinct bacterial (PERMANOVA, R^2^ = 0.165, *p* = 0.014) and fungal (R^2^ = 0.577, *p* = 0.003) community structures between groups. The diseased group exhibited a significant increase in the bacterial phylum Proteobacteria (70.97% vs. 46.27%), primarily driven by the genera *Bibersteinia* and *Pseudomonas*. Fungal communities in the diseased group were dominated by a higher relative abundance of Ascomycota and Basidiomycota, with significant enrichment of *Wallemia* and *Aspergillus*. LEfSe analysis identified *Pseudomonas* and multiple fungal taxa (e.g., *Wallemia*, *Aspergillus*) as biomarkers for the diseased group. PICRUSt2 prediction indicated enrichment of pathways related to carotenoid biosynthesis and sphingolipid metabolism in the diseased state, while FUNGuild analysis suggested a higher abundance of animal/plant pathogen-related fungi. Conclusions: Symptomatic respiratory infections in forest musk deer are associated with significant dysbiosis of the nasopharyngeal microbiome, characterized by the marked enrichment of potential bacterial opportunists (e.g., *Pseudomonas*) and specific fungal taxa (e.g., *Wallemia*, *Aspergillus*), alongside distinct functional shifts in the microbiome. These findings provide the first integrated bacterial–fungal profile of the nasopharyngeal microbiome in this endangered species, and highlight potential microbial biomarkers associated with respiratory disease.

## Simple Summary

The community of tiny organisms, known as the microbiome, live in the nose and throat plays an important role in breathing health in animals. However, little is known about this microbiome in endangered forest musk deer, especially when they are sick. In this study, we examined the bacteria and fungi in the upper respiratory tract of six healthy and six sick forest musk deer. We found that sick forest musk deer had higher levels of a certain type of bacteria and specific fungi, including some linked to disease, which may serve as signs of illness. Functional tests showed changes in metabolic processes and more disease-related fungi in sick forest musk deer. Our findings may help understand illness impacts and support their protection.

## 1. Introduction

The respiratory tract harbors a complex and dynamic microbial ecosystem that plays an integral role in mucosal immunity, pathogen exclusion, and the maintenance of respiratory health [1]. Dysbiosis of this community has been increasingly linked to susceptibility to infectious and inflammatory diseases across diverse animal species, with far-reaching implications for individual welfare, population resilience, and conservation outcomes [2].

The forest musk deer (*Moschus berezovskii*) is a Class I protected species in China [3]. Musk, secreted by male individuals, serves as the sole natural source for valuable traditional medicines and high-grade perfumes, representing significant economic and ecological value [4]. To ensure a sustainable supply of musk, captive farming has been widely adopted. However, intensification of captive farming heightens susceptibility to respiratory tract infections, clinically manifested as nasal discharge, cough, and even pneumonia, often leading to growth retardation, elevated mortality, and substantial economic losses. Current diagnostic approaches rely primarily on clinical observation and the isolation of specific bacterial pathogens [5,6]. These methods are often insufficient, as they may fail to identify subclinical infection, polymicrobial disease, or culture-negative illness. Moreover, they cannot detect the broader ecological disturbances within the respiratory microbiota that precede the onset of overt clinical symptoms. Consequently, a deeper understanding of the microbial dysbiosis associated with these infections is essential for developing effective early warning systems and targeted ecological management strategies.

The upper respiratory tract of mammals harbors a resident microbiome, with the nasopharynx serving as a core ecological niche [7]. As a gateway positioned between the upper and lower airways, this key anatomical site is also an important niche for pathogenic bacterial colonization [8,9]. Numerous studies on humans and livestock have demonstrated that the development of respiratory diseases—such as asthma [10], pneumonia [11], and bovine or ovine respiratory infections [12,13]—is consistently accompanied by significant alterations in the structure, diversity, and function of the nasopharyngeal bacterial community. However, the microbiome is a complex interactive network composed of various organisms, including bacteria, fungi, and viruses [14,15]. Interactions between bacteria and fungi can support host homeostasis, yet when disrupted, may contribute to inflammation and metabolic disorders [16]. In fact, imbalances in fungal communities are also closely associated with respiratory diseases such as chronic sinusitis [17] and asthma [18]. Therefore, focusing solely on the bacterial domain is insufficient for gaining a comprehensive and accurate understanding of the respiratory microbiota and its role in the development of diseases.

To systematically elucidate the microbiological basis of respiratory health and disease, it is essential to conduct integrated research on both bacterial and fungal communities. Although microbiome technology has advanced significantly in the study of respiratory health in other animals, such as bovine [19] and piglets [20], studies on the respiratory microbiome of endangered species like the forest musk deer remain extremely scarce. In this study, we compared the nasopharyngeal microbiomes of healthy and diseased forest musk deer from a breeding facility located in the Qinling Mountains, China. We aimed to: (1) characterize the bacterial and fungal community structures in healthy versus symptomatic individuals, (2) identify differentially abundant taxa and potential microbial biomarkers, (3) predict functional shifts in microbial communities, and (4) explore bacterial–fungal interactions within the respiratory ecosystem. Our findings provide the first comprehensive insight into the respiratory microbiome of forest musk deer and highlight microbial signatures associated with respiratory disease. This work lays a foundation for future studies aimed at microbiome-based health monitoring, disease prevention, and improved management strategies for this endangered species.

## 2. Materials and Methods

### 2.1. Animals and Sample Collection

The Animal breeding facility (34.210832° N, 106.902117° E) is located in Fengxian, Southwest of BaoJi City, Shaanxi Province, China, a region of Qinling mountain at an altitude of 1500 m. All the forest musk deer were separated into two groups: healthy (*n* = 6) (cont) and diseased (*n* = 6) (Res). All animals were 0.5 years of age, with a sex ratio of 1:1 (male:female) in each group. All animals were housed under identical conditions, with ad libitum access to a standardized diet consisting of fresh leaves and clean drinking water daily. No antibiotics or other medications were administered to any of the animals for at least three months prior to sample collection. The stocking density was maintained at one animal per 10 m^2^. The forest musk deer were diagnosed by veterinarians. Physical examination revealed musk deer with nasal discharge, a runny nose or cough clinical symptoms that were diagnosed as diseased, while those with a moist rhinarium, normal appetite, and regular activity were diagnosed as healthy. Nasal and pharyngeal swab samples of sick and healthy forest musk deer with upper respiratory symptoms were collected using sterile disposable nasal swabs, immediately placed on dry ice, and stored in a −80 °C refrigerator for later use.

### 2.2. DNA Extractions, PCR Amplification, 16S rRNA Gene V3–V4 Sequencing and ITS2 Sequencing

The isolation, amplification, and sequencing of bacterial DNA from each sample were performed by LC-Bio Technology Co., Ltd. (Hangzhou, Zhejiang Province, China). The amplicons were obtained using primers 341F (5′-CCTACGGGNGGCWGCAG-3′) and 805R (5′-GACTACHVGGGTATCTAATCC-3′), which covered the V3-V4 region of the bacterial 16S rRNA genes. The ITS2 region of the eukaryotic (fungi) small-subunit rRNA gene was amplified with slightly modified versions of primers ITS1FI2 (5′-GTGARTCATCGAATCTTTG-3′) and ITS2 (5′-TCCTCCGCTTATTGATATGC-3′) [21].

The 5′ ends of the primers were tagged with specific barcodes per sample and sequencing universal primers. PCR amplification was performed in a total volume of 25 μL reaction mixture containing 25 ng of template DNA, 12.5 μL of PCR Premix, 2.5 μL of each primer, and PCR-grade water to adjust the final volume. The PCR conditions to amplify the prokaryotic 16S fragments consisted of an initial denaturation at 98°C for 30 s; 32 cycles of denaturation at 98°C for 10 s, annealing at 54 °C for 30 s, and extension at 72 °C for 45 s; and then final extension at 72 °C for 10 min. The PCR products were confirmed with 2% agarose gel electrophoresis. Throughout the DNA extraction process, ultrapure water, instead of a sample solution, was used to exclude the possibility of false-positive PCR results as a negative control. The PCR products were purified by AM Pure XT beads (Beckman Coulter Genomics, Danvers, MA, USA) and quantified by Qubit (Invitrogen, Carlsbad, CA, USA). The amplicon pools were prepared for sequencing and the size and quantity of the amplicon library were assessed on Agilent 2100 Bioanalyzer (Agilent, Santa Clara, CA, USA) and with the Library Quantification Kit for Illumina (Kapa Biosciences, Woburn, MA, USA), respectively. The libraries were sequenced on the NovaSeq6000 PE250 platform (Illumina, San Diego, CA, USA).

### 2.3. Bioinformatics Analysis

Raw paired-end sequencing data were processed using QIIME2 (version 2019.07.) [22]. Adapters and primer sequences were trimmed using cutadapt (integrated via QIIME2 cutadapt plugin). Forward and reverse reads were merged using vsearch (via QIIME2 vsearch merge-pairs command). Low-quality sequences were filtered based on Q-score thresholding (QIIME2 quality-filter q-score). Chimeric sequences were filtered out using Vsearch software (v2.3.4). Clean reads were then conducted on feature classification to output ASVs (amplicon sequence variants) by dada2, and the ASVs with counts less than 2 in all samples were filtered. Species annotation was performed based on the ASV (feature) Sequences of bacteria annotated using the SILVA (v138.2, https://www.arb-silva.de/documentation/release-1382/, accessed on 23 July 2025) [23] database and the NT-16S database. Sequences of fungi alignment of species annotation was performed by the QIIME2 plugin feature classifier, and the alignment database was the RDP and UNITE (2019, https://unite.ut.ee/) [24] databases. For both annotations, a confidence threshold of 0.7 was applied to retain high-confidence taxonomic assignments. QIIME 2 was applied to analyze the alpha diversity and beta diversity (based on unweighted UniFrac distance), and the corresponding plots were generated by R (v3.5.2). LEfSe (Linear discriminant analysis effect size) [25] was performed to detect differentially abundant taxa across groups using the default parameters. Wilcoxon rank-sum test and PERMANOVA were employed to examine the significance of intergroup differences. Microbial functional profiles for bacteria and fungi were predicted with PICRUSt2 (version2.6.2) [26] and FUNGuild (ITS)(version 0.3.0) [27], respectively. A co-occurrence network at the key genus level was constructed based on Spearman correlation analysis between bacterial and fungal taxa. The heatmap was drawn based on R (https://www.r-project.org/) on the OmicStudio platform (https://www.omicstudio.cn/tool). For all statistical analyses, a threshold of *p < 0.05 was considered statistically significant.

## 3. Results

### 3.1. Overview of Sequencing Data and Microbial Community Diversity

Bacterial 16S rRNA gene V3–V4 sequencing of the 12 samples generated a total of 994,711 raw tags. After quality control, 887,505 effective tags were obtained, with a mean (±standard deviation) of 73,958.75 ± 5897.09 per sample (Table S1). For fungal ITS sequencing, 1,002,744 raw tags were obtained, yielding 942,958 effective tags after quality control, with a mean of 78,579.83 ± 2756.71 per sample (Table S2). Notably, the sequencing depth coverage for all samples exceeded 99.5%, indicating that the sequencing depth was sufficient to represent the true microbial composition of the samples.

**Figure 1:**
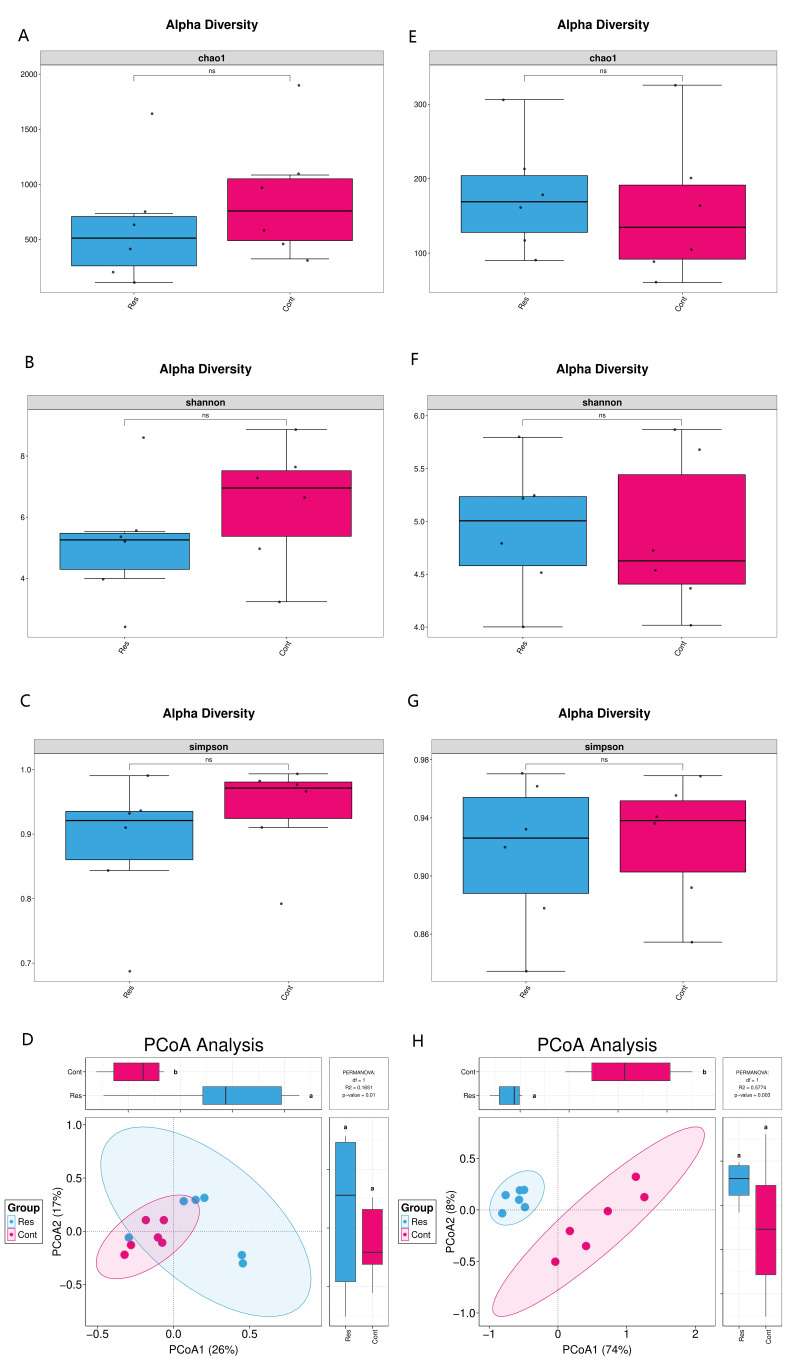
Analysis of nasopharyngeal microbial community diversity between the Res group and Cont group in forest musk deer. (**A**) Boxplot of the Chao1 index for bacterial communities, representing species richness; (**B**) Boxplot of the Shannon index for bacterial communities, representing species diversity. No significant differences were observed in either index between the Res (red) and Cont (blue) groups. (**C**) Boxplot of the Simpson index for bacterial communities, representing species dominance and diversity (**E**) Boxplot of Chao1 index and (**F**) boxplot of Shannon index and boxplot of the Simpson index. (**G**) for fungal communities, indicating no significant differences between groups. (**D**) PCoA analysis of bacterial community. The community structures of the two groups of samples were significantly separated (PERMANOVA, R^2^ = 0.1651, *p* = 0.014). (**H**) PCoA analysis of fungal community. There was a significant difference in the community structures between the two groups of samples (PERMANOVA, R^2^ = 0.5774, *p* = 0.003). The Res group is marked in red and the control group (Cont) in blue. ns, not significant.

Analysis of microbial alpha diversity based on 16S rRNA gene sequencing revealed that no significant differences were observed in the Chao1 index (Figure 1A), Shannon index (Figure 1B), and Simpson index (Figure 1C) of nasopharyngeal microbiota in the Res groups compared with the Cont groups. Similarly, ITS sequencing (fungal) analysis showed no significant differences in the Chao1 index (Figure 1E) or Shannon index (Figure 1F), and Simpson index (Figure 1G) between the two groups. Rarefaction curves indicated that sequencing depth was sufficient to capture the majority of microbial diversity in all samples for both 16S and ITS datasets, with curves reaching a plateau (Supplementary Figure S1). These results indicate that the richness and evenness of the microbial community in the nasopharynx of forest musk deer did not differ substantially between the Res and Cont groups. Principal coordinate analysis (PCoA) demonstrated a significant separation between the two groups at the bacterial community level (PERMANOVA, R^2^ = 0.1651, *p* = 0.014; Figure 1D). At the fungal community level, the separation trend between the groups was even more pronounced (PERMANOVA, R^2^ = 0.5774, *p* = 0.003; Figure 1H). These findings demonstrated that the microbial community structures differed significantly between diseased and healthy forest musk deer.

### 3.2. Microbial Community Composition and Differential Taxon Analysis

In this study, a total of 29 bacterial phyla, 61 classes, 139 orders, 243 families, 329 genera, and 573 species were identified. Additionally, 8 fungal phyla, 32 classes, 89 orders, 195 families, 329 genera, and 488 species were detected.

**Figure 2:**
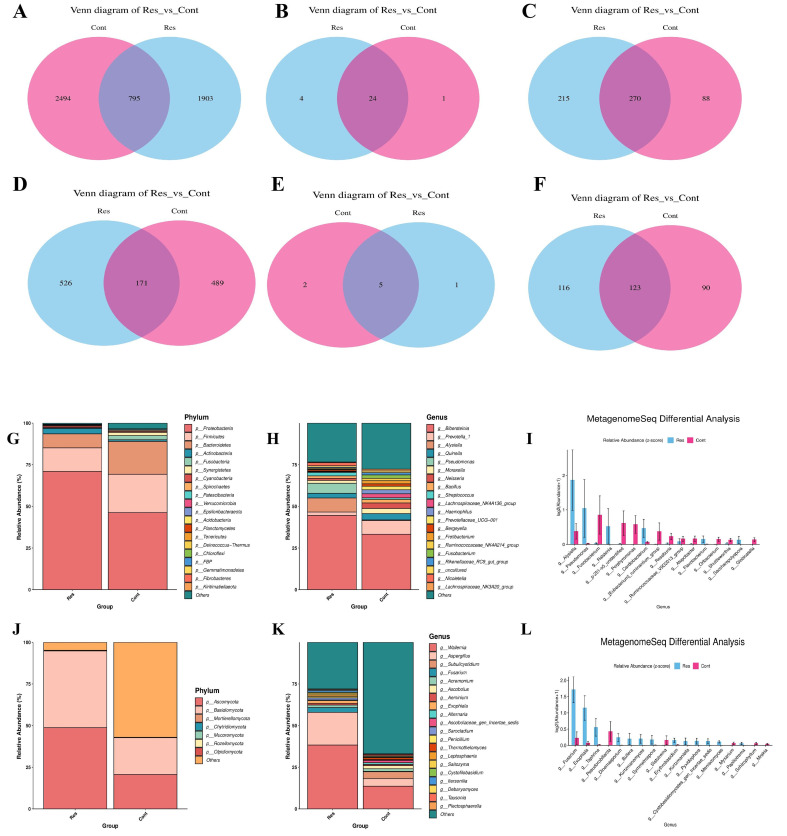
Analysis of shared taxa, species composition, and differential analysis of nasopharyngeal microbiota between the Res and Cont groups of forest musk deer; (**A**–**C**) Bacterial 16S rRNA gene V3–V4 sequencing analysis: Venn diagrams show at the ASV level (**A**), phylum level (**B**), and genus level (**C**), showing the number of shared and unique taxa between the two groups across different taxonomic ranks. (**D**–**F**) Fungal ITS sequencing analysis: Venn diagrams show at the ASV level (**D**), phylum level (**E**), and genus level (**F**), displaying the number of shared and unique taxa of fungi across different taxonomic ranks. (**G**,**H**) Bacterial community composition: Stacked bar charts at the ASV level (**G**) and genus level (**H**). (**J**,**K**) Fungal community composition: Stacked bar charts at the phylum level (**J**) and genus level (**K**). (**I**) Bacterial differential analysis: Differential analysis of bacterial genera based on MetagenomeSeq. (**L**) Fungal differential analysis: Differential analysis of fungal genera based on MetagenomeSeq.

Venn analysis revealed distinct “shared + unique” patterns across taxonomic levels in both bacterial and fungal datasets (Figure 2). For the bacterial community, the Cont group harbored 2494 unique amplicon sequence variants (ASVs), compared with 1903 unique ASVs in the Res groups, with 795 ASVs shared between the two groups (Figure 2A). At the phylum level, only 1 unique phylum was detected in the Cont group versus 4 unique phyla in the Res group, while 24 phyla were shared (Figure 2B). At the genus level, 88 and 215 unique genera were identified in the Cont and Res groups, respectively, with an additional 270 genera shared between groups (Figure 2C). In the fungal community, the Cont and Res groups contained 489 and 526 unique ASVs, respectively, with 171 ASVs shared (Figure 2D). At the phylum level, the Cont group had 2 unique phyla, the Res group had 1 unique phylum, and 5 phyla were shared (Figure 2E). At the genus level, 99 and 116 unique genera were observed in the Cont and Res groups, respectively, with 123 genera being common (Figure 2F). Collectively, these results indicate that the Res and Cont groups exhibit significant differences in microbial community composition.

Further analysis of the bacterial community composition at the phylum level (Figure 2G) revealed that the predominant phyla in both groups were Proteobacteria, Firmicutes, and Bacteroidetes. However, their relative abundances differed significantly between groups. In the Res group, the relative abundance of Proteobacteria (70.97%) was markedly higher than that in the Cont group (46.27%), whereas Firmicutes (14.15% vs. 22.99%) and Bacteroidetes (8.40% vs. 19.72%) were lower. Furthermore, the relative abundance of Actinobacteria was higher in the Res group (3.31%) than in the Cont group (0.99%). The relative abundance of Fusobacteria also differed significantly (*p* < 0.05), with lower levels observed in the Res groups. At the genus level (Figure 2H), among the top 20 genera, only four—*Bibersteinia*, *Alysiella*, *Pseudomonas*, and *Streptococcus*—were enriched in the Res group; specifically, *Bibersteinia* was significantly more abundant in the Res group (44.59%) than in the Cont group (33.28%). The relative abundance of *Alysiella* increased from 0.38% to 8.43%, while that of *Streptococcus* rose from 1.04% to 2.14%. Most strikingly, *Pseudomonas* displayed a substantial emergent enrichment trend, with its relative abundance surging from 0.01% to 6.05%. Conversely, *Prevotella_1* and *Haemophilus* were depleted in the Res group; for example, the relative abundance of *Prevotella_1* dropped from 8.19% to 2.01%. Collectively, these data demonstrate a significant structural divergence in nasopharyngeal microbiota between the two groups at the genus level, with differential enrichment of key taxa—particularly *Bibersteinia* and *Pseudomonas*—likely representing the primary drivers of this community differentiation.

Fungal community analysis (Figure 2J) revealed that Ascomycota and Basidiomycota were the dominant phyla in both groups, with their relative abundances differing significantly between groups. Specifically, the relative abundance of Ascomycota was markedly higher in the Res group (48.80%) than in the Cont group (20.65%); similarly, Basidiomycota exhibited a higher relative abundance in the Res group (46.04%) compared to the Cont group (22.09%). At the genus level (Figure 2K), the fungal community in the Res group was dominated by *Wallemia* (38.34%) and *Aspergillus* (19.62%) with a relatively concentrated community structure. In contrast, the Cont group was primarily composed of unclassified fungi (~57%) with no clearly dominant genera, suggesting a more dispersed community structure.

MetagenomeSeq differential analysis (Figure 2I) showed that, for the bacterial community, the relative abundances of genera including *Alysiella*, *Pseudomonas*, and *Ralstonia* (after z-score normalization) were significantly higher in the Res group, whereas *Fusobacterium* was more abundant in the Cont group. For the fungal community (Figure 2L), the Res group had significantly elevated relative abundances of genera such as *Fusarium*, *Exophiala*, and *Taphinia*. Collectively, these results demonstrate distinct disparities in fungal community composition between the two groups, with taxa including *Fusarium* representing the core discriminatory features that distinguish the Res and Cont groups.

### 3.3. LEfSe Analysis of Bacteria and Fungi

**Figure 3:**
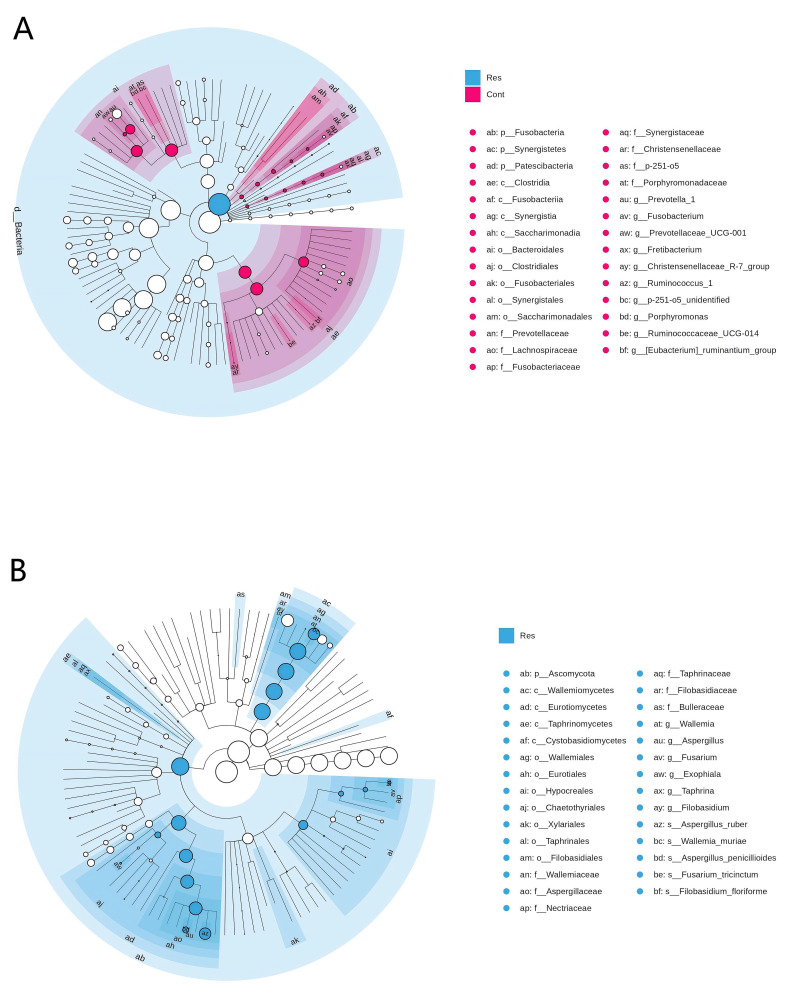
LEfSe analysis of nasopharyngeal microbiota between the Cont and Res groups of forest musk deer (**A**) LEfSe analysis of bacterial communities. Red nodes/branches represent bacterial taxa (from phylum to genus) with significantly higher abundance in the Cont group; white nodes represent taxa with no significant difference between groups. (**B**) LEfSe analysis of fungal communities. Blue nodes/branches represent fungal taxa (from phylum to species) with significantly higher abundance in the Res group; white nodes represent taxa with no significant difference between groups.

Based on LEfSe analysis (LDA > 3.0, *p* < 0.05), several microbial taxa were identified as significantly differentially abundant between the groups (Figure 3). Given the exploratory nature of LEfSe and the limited sample size, these findings should be interpreted as hypothesis-generating. For the bacterial community (Figure 3A), the Cont group (red nodes) exhibited enrichment across multiple taxonomic levels from phylum to genus, including *Fusobacteria*, *Fusobacteriia*, *Fusobacteriales*, and *Fusobacterium*, among others. In contrast, the Res group (blue nodes) showed only limited enrichment, which may imply divergence in patterns of dominant bacterial taxa between the two groups. In the fungal community (Figure 3B), the Res group displayed potential enrichment of several taxa, such as Ascomycota, Wallemiomycetes, *Wallemia*, *Aspergillus*, as well as species including *Aspergillus_ruber* and *Wallemia_muriae*, whereas no significant enrichment was observed in the Cont group. These results suggest potential compositional differences in both bacterial and fungal communities of the nasopharynx between the two groups; further validation in larger independent cohorts is required.

### 3.4. KEGG Pathway and Fungal Guild Discrepancies in Forest Musk Deer Nasopharyngeal Microbiome

**Figure 4:**
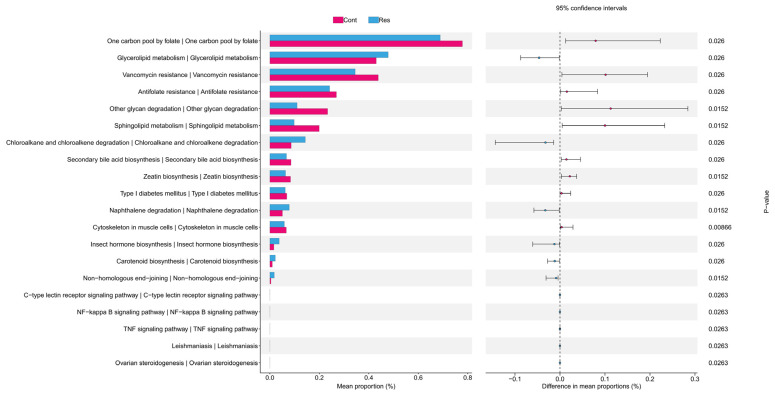
PICRUSt2-based functional prediction of microbial functional differences in KEGG level 3 pathways.

Using PICRUSt2 for functional prediction and STAMP for statistical analysis, we compared the microbial functional discrepancies between the two groups at the KEGG pathway Level 3 (Figure 4). Antibiotic resistance-related pathways exhibited significant inter-group divergence: the abundances of vancomycin resistance and antifolate resistance pathways were higher in the Cont group than in the Res group, indicating group-specific resistance profiles that may be attributed to environmental factors or intervention conditions. At the metabolic level, secondary bile acid biosynthesis was enriched in the Cont group, whereas carotenoid biosynthesis was overrepresented in the Res group. Moreover, significant variations were observed in sphingolipid metabolism and the one-carbon pool by folate pathways. For disease- and immunity-associated pathways, the Res group showed lower abundance of genes linked to type I diabetes mellitus. Differential enrichment of the NF-κB and C-type lectin receptor signaling pathways further supported the potential role of the microbiome in host immune regulation. Collectively, these divergent pathways reveal systematic differentiation in the functional profiles of the two microbial communities, providing critical insights into the adaptive changes in microbial functions under specific conditions and their interactive mechanisms with the host.

**Figure 5:**
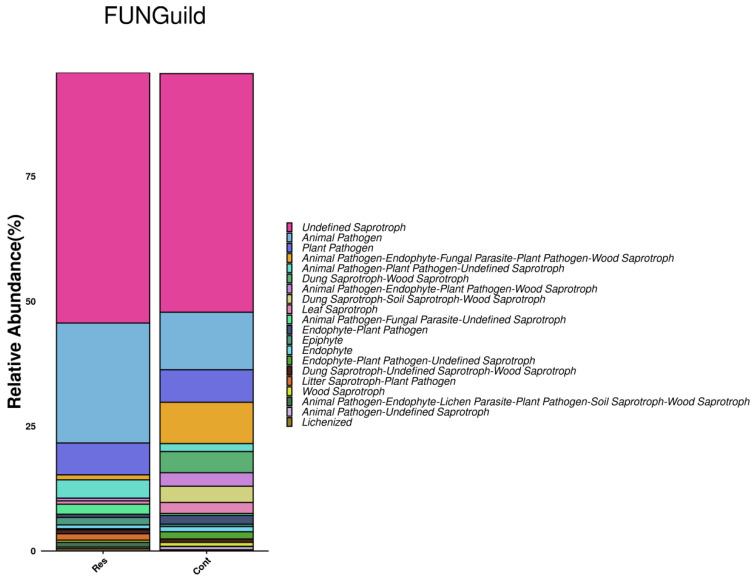
Comparison of relative abundances of fungal functional guilds between the Res and Cont groups based on FUNGuild.

As shown in Figure 5, fungal functional annotation via FUNGuild indicated a marked contrast between the two groups: the Res group harbored a relatively higher proportion of taxa linked to animal pathogens and plant pathogens, while saprotrophic and endophytic functional groups were more prevalent in the normal group.

### 3.5. Correlation Analysis Between Fungi and Bacteria Genera

**Figure 6:**
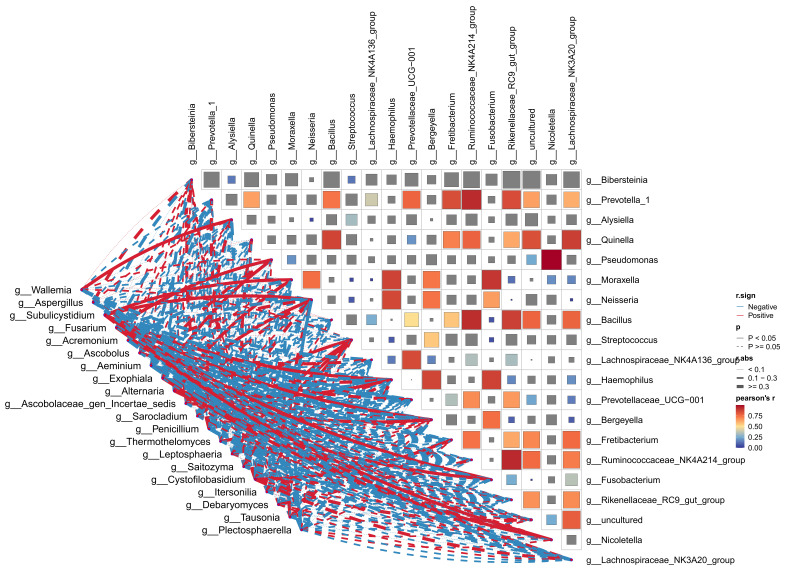
Pearson correlation heatmap and association network of microbial taxa. The color gradient in the heatmap represents Pearson’s correlation coefficient (*r*): Blue indicates negative correlation, red indicates positive correlation, and light colors/white represent weak correlation. In the network diagram, red lines correspond to positive correlations; blue lines correspond to negative correlations. The line thickness is positively correlated with the absolute value of the correlation strength (|*r*|). Solid lines represent a statistically significant correlation (*p* < 0.05), while dashed lines represent non-significant correlations (*p* ≥ 0.05).

A total of 81 paired combinations, involving 9 fungal genera (e.g., *Wallemia*, *Aspergillus*, *Subulicystidium*) and 10 bacterial genera (e.g., *Bibersteinia*, *Prevotella_1*, *Alysiella*), were subjected to the cross-kingdom correlation analysis. Association patterns among all combinations were visualized using correlation heatmaps and network diagrams (Figure 6). Only three combinations exhibited statistical significance (*p* < 0.05), all of which were strongly positive (|*r*| ≥ 0.3). Specifically, *Ascobolus* and *Alysiella* exhibited the strongest positive correlation (*r* = 0.922, *p* = 0.00002), suggesting a highly synchronous abundance pattern and potential synergistic interaction. Additionally, two other pairs (*Aeminium* vs. *Pseudomonas*, r = 0.705, *p* = 0.0105; *Aspergillus* vs. *Moraxella*, r = 0.704, *p* = 0.0105) indicate strong positive associations, which may reflect functional or metabolic linkages within the microbial community.

Although several combinations were non-significant (*p* ≥ 0.05), they still displayed considerable correlation strength (|*r*| ≥ 0.3), suggesting potential trends that warrant further investigation. These included the negative correlation between *Aspergillus* and *Bibersteinia* (*r* = −0.396), positive correlations between *Subulicystidium* and *Bibersteinia* (*r* = 0.468) as well as *Alternaria* and *Bibersteinia* (*r* = 0.511), and the negative correlation between *Fusarium* and *Streptococcus* (*r* = −0.395). The absence of statistical significance for these combinations may be attributed to limited sample size or environmental interference; however, their substantial correlation magnitude implies potential interspecific interactions that merit further investigation.

## 4. Discussion

This study provides a comprehensive characterization of the nasopharyngeal microbiota (bacterial and fungal) in forest musk deer (*Moschus berezovskii*), revealing significant structural and functional shifts associated with upper respiratory tract (URT) disease. Our findings contribute to the growing evidence that the respiratory microbiome is a dynamic ecosystem, the dysbiosis of which is intricately linked to host health.

Although no significant differences were observed in α-diversity (richness and evenness) between healthy (Cont) and diseased (Res) groups, β-diversity analysis demonstrated a clear distinction in bacterial and fungal community structures between the two groups. These findings are consistent with previous reports on respiratory diseases in humans and other animals. For example, in bovine respiratory disease (BRD), microbial composition—rather than α-diversity—has been shown to effectively differentiate healthy from diseased individuals [13,28,29]. Similar alterations in microbial community structure have been documented in children with respiratory symptoms [30] and in ovine [12]. Notably, in this study, the structural shift was more pronounced in the fungal community, as indicated by a higher *R*^2^ value in PERMANOVA. This underscores the underexplored potentially critical role of fungal microbiota in respiratory health—a perspective that is increasingly recognized in recent research involving humans and animals [16,31].

At the taxonomic level, a “shared + unique” distribution pattern was evident between the Res and Cont groups across all ranks for both bacteria and fungi. Such patterns are commonly reported in mammalian respiratory microbiota studies [32] and likely reflect adaptive restructuring in response to host physiological changes. Shared taxa may maintain baseline nasopharyngeal functions, whereas unique taxa could be linked to disease-related processes. Specifically, at the phylum level, the Res group showed a significant increase in Proteobacteria, accompanied by decreases in Firmicutes and Bacteroidetes. This pattern has been reported in various respiratory diseases [28,33] and may represent a conserved signature of dysbiosis in respiratory illness. Furthermore, several bacterial genera, including *Bibersteinia*, *Pseudomonas*, and *Alysiella*, were significantly enriched in the Res group. Among them, *Bibersteiniatrehalosi* has been confirmed as a respiratory pathogen in ruminants and can cause pneumonia [34,35]; its high abundance suggests that it may play a pathogenic role in the respiratory symptoms of forest musk deer. *Pseudomonas*, as a common opportunistic pathogen, showed a “zero-to-detectable” increase in the Res group, consistent with the microbiota dysbiosis in human cystic fibrosis (CF) lung infections and Ventilator-Associated Pneumonia [36,37]. This may reflect weakened host immune defense or mucosal environment alterations following opportunistic colonization. Conversely, Fusobacteria and the genus *Fusobacterium* were reduced in the Res group, possibly reflecting changes in the local inflammatory state. The genus *Fusobacterium* is often associated with oral and respiratory infections [38,39], and its change in abundance further suggests a shift in the inflammatory status of the nasopharynx.

The fungal community also underwent marked restructuring in diseased individuals, with *Wallemia* and *Aspergillus* as dominant genera. Fungi are increasingly recognized as important contributors to mucosal disorders, capable of modulating bacterial behavior and host immune responses [40,41]. For instance, in a neonatal intestinal model, fungal overgrowth was shown to exacerbate subsequent allergic airway inflammation [42]. Similarly, elevated fungal abundance has been reported in horses with nasopharyngeal scarring syndrome [43]. In the present study, LEfSe analysis highlighted *Wallemia* and *Aspergillus* as potential biomarkers distinguishing the Res from the Cont group. Notably, *Aspergillus* is a well-established respiratory fungal pathogen associated with a spectrum of diseases ranging from allergy to invasive infection [44,45]. Recent studies indicate that members of the genus *Wallemia*, such as *Wallemiamellicola*, can exacerbate asthma via the Dectin-2/CARD9 signaling pathway [46,47], providing a mechanistic insight into the link between *Wallemia* enrichment and upper respiratory symptoms in forest musk deer. Functional prediction using FUNGuild revealed a higher relative abundance of fungi classified as “animal pathogens” in the diseased group, supporting the potential pathogenic role of these fungi at a functional level. However, it is important to note that approximately 57% of the fungal community in the healthy group remained unclassified at the genus level, primarily due to the reliance on the UNITE database (2019) and the inherent limitations of ITS-based taxonomic assignment for poorly characterized environmental or host-associated fungi. This high proportion of unassigned taxa represents a notable limitation, as it may obscure certain differentially abundant taxa and potentially bias functional predictions derived from annotation-dependent tools such as FUNGuild. Nevertheless, despite this limitation, the robust identification of *Wallemia* and *Aspergillus*—both well-documented respiratory pathogens with established mechanistic links to airway inflammation—as core discriminatory taxa suggests that the key microbial signals driving community differentiation are sufficiently strong to be captured

Functional prediction using PICRUSt2 revealed systematic differences in metabolic and immune-related pathways between groups. Notably, pathways associated with antibiotic resistance were more abundant in Cont groups; the enrichment of antibiotic resistance genes is often associated with environmental antibiotic exposure or microbial resistance evolution, possibly reflecting an adaptive defense mechanism of the nasopharyngeal microbiota in healthy forest musk deer against environmental stress. Mammalian microbiomes harbor a substantial reservoir of antibiotic resistance genes, whose distribution is closely linked to the host’s living environment, and some genes pose a risk of cross-host transmission [48]. In contrast, significant metabolic change was observed in the Res group: a reduction in the “Secondary bile acid biosynthesis” pathway and an increase in the “Carotenoid biosynthesis” pathway. Bile acids are potent signaling molecules that modulate host inflammation and microbial composition [49,50], and alterations in their microbial metabolism may affect local immune responses. Meanwhile, microbial carotenoid production exhibits antioxidant and anti-inflammatory properties [51,52]. Its upregulation may represent a microbial community response to oxidative stress at the mucosal level in inflammatory upper respiratory tract infections. Differential enrichment of immune-related pathways, such as the NF-κB and C-type lectin receptor (CLR) signaling, further underscores the close interaction between the nasopharyngeal microbiota and the host immune state [53,54].

Correlation network analysis provided preliminary insights into the complex cross-domain interactions between bacteria and fungi within the nasopharyngeal microbiota of forest musk deer. This study identified a limited number of strong positive correlations, such as those between the fungal genus *Ascobolus* and the bacterial genus *Alysiella*, and between *Aspergillus* and *Moraxella*. Bacterial–fungal interactions (BFI) represent a central aspect of microbial ecology, encompassing diverse forms such as symbiosis, antagonism, and competition [31,55,56]. These cross-domain interactions can significantly influence microbial community stability, virulence expression, and host immune outcomes [57,58]. For instance, in cystic fibrosis airways, co-colonization of *Candida albicans* and *Pseudomonas aeruginosa* can modulate disease progression through a complex interplay [59]. Similarly, in mouse models, co-infection with *C. albicans* and *Staphylococcus aureus* leads to more severe inflammation and tissue damage compared to mono-infection [60]. These examples illustrate how specific bacterial–fungal consortia may act synergistically to compromise host defenses. The observed association patterns in this study suggest that in the musk deer nasopharyngeal environment, *Aspergillus* and *Moraxella* may form a similar pathogenic cross-kingdom alliance, potentially sustaining and exacerbating dysbiosis through metabolic crosstalk and immune modulation [61]. It should be noted, however, that this network analysis is based on a limited sample size, and the identified correlations remain preliminary evidence.

## 5. Conclusions

In summary, this study delineates a distinct nasopharyngeal dysbiosis signature associated with respiratory disease in forest musk deer, characterized by a bacterial shift toward Proteobacteria, expansion of specific bacterial (e.g., *Bibersteinia*, *Pseudomonas*) and fungal taxa (e.g., *Wallemia*, *Aspergillus*), and predicted alterations in community function—including antioxidant synthesis and immune modulation pathways. These alterations are likely linked to the clinical disease phenotype in the host. Notably, we report for the first time a marked enrichment of the fungal genus *Wallemia* in forest musk deer with respiratory symptoms, offering a new perspective on the role of fungi in respiratory health among non-model animals. While these findings reveal associations between microbial alterations and disease status, longitudinal studies and intervention trials are needed to determine whether microbiome monitoring can serve as a predictive tool for disease risk or as an indicator for management interventions in this vulnerable species.

### Limitations of the Study

Several limitations of this study should be acknowledged. First, the cross-sectional design precludes causal inference regarding whether the observed microbial shifts precede or are induced by the disease status. Second, the relatively modest sample size may compromise statistical power, potentially masking biologically relevant microbial associations. Third, disease classification was based primarily on clinical signs (nasal discharge and coughing) assessed by experienced veterinarians, without inclusion of objective clinical parameters such as body temperature or blood tests. This decision was made considering the economic burden and welfare implications of invasive sampling in valuable forest musk deer, particularly given that this study aimed to preliminarily characterize nasopharyngeal bacterial and fungal profiles under initial clinical presentations. We acknowledge that this approach may introduce classification bias given the phenotypic heterogeneity of respiratory diseases. Future studies would benefit from incorporating comprehensive clinical and laboratory assessments to refine disease phenotyping and strengthen the reliability of microbial comparative analyses. Finally, predicted functional alterations are solely derived from bioinformatic inferences and thus require validation via targeted metabolomic experiments.

## Data Availability

Raw sequencing reads have been deposited in the China National Center for Bioinformatics (CNCB) under the accession number PRJCA055982.

## Supplementary Materials

The following supporting information can be downloaded at: https://www.mdpi.com/article/10.3390/biology15070587/s1, Table S1. Summary of 16S rRNA gene V3-V4 amplicon sequencing data quality metrics. Table S2. Summary of valid sequencing data for ITS amplicons. Figure S1. Rarefaction curve of 16S rRNA gene sequencing. A: Rarefaction curve of 16S rRNA gene sequencing. B: Coverage curve of ITS sequence sequencing.

## References

[1] Man W.H., de Steenhuijsen Piters W.A., Bogaert D., Debby B. (2017). The microbiota of the respiratory tract: Gatekeeper to respiratory health. Nat. Rev. Microbiol..

[2] Trevelline B.K., Fontaine S.S., Hartup B.K., Kohl K.D. (2019). Conservation biology needs a microbial renaissance: A call for the consideration of host-associated microbiota in wildlife management practices. Proc. Biol. Sci..

[3] Yang Q., Meng X., Xia L., Feng Z. (2003). Conservation status and causes of decline of musk deer (*Moschus* spp.) in China. Biol. Conserv..

[4] Shuquan L., Zhixin L., Ge Y., Afzal S.S., Saeed A., Taolei S. (2021). Chemical compositions and pharmacological activities of natural musk (*Moschus*) and artificial musk: A review. J. Ethnopharmacol..

[5] Zhao W., Tian Q., Luo Y., Wang Y., Yang Z.X., Yao X.P., Cheng J.G., Zhou X., Wang W.Y. (2017). Isolation, Identification, and Genome Analysis of Lung Pathogenic *Klebsiella pneumoniae* (Lpkp) in Forest Musk Deer. J. Zoo Wildl. Med..

[6] Luo X., Wang P., Cheng J.G., Luo Y., Dai L., Zhou X., Zou L.K., Li B., Xiao J.J. (2016). Characterization of Virulence Genes and Antimicrobial Resistance of Lung Pathogenic *Escherichia coli* Isolates in Forest Musk Deer (*Moschus berezovskii*). J. Zoo Wildl. Med..

[7] Cleary D.W., Clarke S.C. (2017). The nasopharyngeal microbiome. Emerg. Top. Life Sci..

[8] Huang Y.J. (2017). Nasopharyngeal Microbiota: Gatekeepers or Fortune Tellers of Susceptibility to Respiratory Tract Infections?. Am. J. Respir. Crit. Care Med..

[9] Matthew F., James D. (2021). The microbiome of the nasopharynx. J. Med. Microbiol..

[10] van Beveren G.J., de Steenhuijsen Piters W.A.A., Boeschoten S.A., Louman S., Chu M.L., Arp K., Fraaij P.L., de Hoog M., Buysse C., van Houten M.A. (2024). Nasopharyngeal microbiota in children is associated with severe asthma exacerbations. J. Allergy Clin. Immunol..

[11] Dietl B., Henares D., Cuchi E., Blanco-Fuertes M., Rajadell M., Brotons P., Lluansi A., Boix-Palop L., Calbo E., Munoz-Almagro C. (2025). Differential nasopharyngeal microbiota patterns: A comparative study of pneumococcal pneumonia, COVID-19, and healthy adults. J. Infect..

[12] Jiang H., Yang L., Duan S., Wu R., Li M., Liu B., Zhu Y., Li J. (2024). Analysis of nasopharyngeal microbiota revealing microbial disturbance associated with ovine respiratory complex. Res. Vet. Sci..

[13] Holman D.B., McAllister T.A., Topp E., Wright A.D., Alexander T.W. (2015). The nasopharyngeal microbiota of feedlot cattle that develop bovine respiratory disease. Vet. Microbiol..

[14] Rowan-Nash A.D., Korry B.J., Mylonakis E., Belenky P. (2019). Cross-Domain and Viral Interactions in the Microbiome. Microbiol. Mol. Biol. Rev..

[15] Deveau A., Bonito G., Uehling J., Paoletti M., Becker M., Bindschedler S., Hacquard S., Herve V., Labbe J., Lastovetsky O.A. (2018). Bacterial-fungal interactions: Ecology, mechanisms and challenges. FEMS Microbiol. Rev..

[16] Li D., Zhang D.Y., Chen S.J., Lv Y.T., Huang S.M., Chen C., Zeng F., Chen R.X., Zhang X.D., Xiong J.X. (2025). Long-term alterations in gut microbiota following mild COVID-19 recovery: Bacterial and fungal community shifts. Front. Cell. Infect. Microbiol..

[17] Yang H., Simpson C.A., Srivastava M., Bera A., Cappelletti M., Suh J.D., Wang M.B., Beswick D.M., Maxim T., Basak S.K. (2025). Biodiversity of the Bacterial and Fungal Microbiome and Associated Inflammatory Cytokine Profile in Chronic Rhinosinusitis. Int. Forum Allergy Rhinol..

[18] Perez-Losada M., Castro-Nallar E., García-Huidobro J., Boechat J.L., Delgado L., Rama T.A., Oliveira M. (2024). The nasal mycobiome of individuals with allergic rhinitis and asthma differs from that of healthy controls in composition, structure and function. Front. Microbiol..

[19] Okamura S., Fukuda A., Usui M. (2024). Rapid detection of causative bacteria including multiple infections of bovine respiratory disease using 16S rRNA amplicon-based nanopore sequencing. Vet. Res. Commun..

[20] Zhang J., Shi K., Wang J., Zhang X., Zhao C., Du C., Zhang L. (2020). Effects of respiratory disease on Kele piglets lung microbiome, assessed through 16S rRNA sequencing. Vet. World.

[21] Karlsson I., Friberg H., Steinberg C., Persson P. (2014). Fungicide effects on fungal community composition in the wheat phyllosphere. PLoS ONE.

[22] Bolyen E., Rideout J.R., Dillon M.R., Bokulich N.A., Abnet C.C., Al-Ghalith G.A., Alexander H., Alm E.J., Arumugam M., Asnicar F. (2019). Reproducible, interactive, scalable and extensible microbiome data science using QIIME 2. Nat. Biotechnol..

[23] Quast C., Pruesse E., Yilmaz P., Gerken J., Schweer T., Yarza P., Peplies J., Glockner F.O. (2013). The SILVA ribosomal RNA gene database project: Improved data processing and web-based tools. Nucleic Acids Res..

[24] Nilsson R.H., Larsson K.-H., Taylor A.F.S., Bengtsson-Palme J., Jeppesen T.S., Schigel D., Kennedy P., Picard K., Glöckner F.O., Tedersoo L. (2019). The UNITE database for molecular identification of fungi: Handling dark taxa and parallel taxonomic classifications. Nucleic Acids Res..

[25] Segata N., Izard J., Waldron L., Gevers D., Miropolsky L., Garrett W.S., Huttenhower C. (2011). Metagenomic biomarker discovery and explanation. Genome Biol..

[26] Douglas G.M., Maffei V.J., Zaneveld J.R., Yurgel S.N., Brown J.R., Taylor C.M., Huttenhower C., Langille M.G.I. (2020). PICRUSt2 for prediction of metagenome functions. Nat. Biotechnol..

[27] Tanunchai B., Ji L., Schroeter S.A., Wahdan S.F.M., Hossen S., Delelegn Y., Buscot F., Lehnert A.S., Alves E.G., Hilke I. (2023). FungalTraits vs. FUNGuild: Comparison of Ecological Functional Assignments of Leaf- and Needle-Associated Fungi Across 12 Temperate Tree Species. Microb. Ecol..

[28] Zeineldin M., Lowe J., de Godoy M., Maradiaga N., Ramirez C., Ghanem M., El-Raof Y.A., Aldridge B. (2017). Disparity in the nasopharyngeal microbiota between healthy cattle on feed, at entry processing and with respiratory disease. Vet. Microbiol..

[29] Timsit E., Holman D.B., Hallewell J., Alexander T.W. (2016). The nasopharyngeal microbiota in feedlot cattle and its role in respiratory health. Anim. Front..

[30] Missa K.F., Diallo K., Bla K.B., Tuo K.J., Gboko K.D.T., Tiémélé L.S., Ouattara A.F., Gragnon B.G., Ngoi J.M., Wilkinson R.J. (2024). Association of symptomatic upper respiratory tract infections with the alteration of the oropharyngeal microbiome in a cohort of school children in Côte d’Ivoire. Front. Microbiol..

[31] Zhang M., Zhang Y., Zhao Z., Deng F., Jiang H., Liu C., Li Y., Chai J. (2025). Bacterial–Fungal Interactions: Mutualism, Antagonism, and Competition. Life.

[32] Bond S.L., Timsit E., Workentine M., Alexander T., Leguillette R. (2017). Upper and lower respiratory tract microbiota in horses: Bacterial communities associated with health and mild asthma (inflammatory airway disease) and effects of dexamethasone. BMC Microbiol..

[33] Qing X., Yu X., Yukun H., Yan Y., Guiju F., Wenyi Y., Jianhui W., Jiwei L., Lili Z., Xinyu D. (2023). Lung microbiome and cytokine profiles in different disease states of COPD: A cohort study. Sci. Rep..

[34] Brown S.E., Bycroft K.A., Adam K., Collett M.G. (2020). Acute fibrinous pleuropneumonia and septicaemia caused by *Bibersteinia trehalosi* in neonatal calves in New Zealand. N. Z. Vet. J..

[35] Guo R., Xu M., Yang K., Gao T., Zhu J., Liu W., Yuan F., Liu Z., Li C., Wu Q. (2024). Isolation, identification and characteristics of *Bibersteinia trehalosi* from goat. Microb. Pathog..

[36] Lopes S.P., Azevedo N.F., Pereira M.O. (2015). Microbiome in cystic fibrosis: Shaping polymicrobial interactions for advances in antibiotic therapy. Crit. Rev. Microbiol..

[37] Bustos I.G., Martín-Loeches I., Acosta-González A., Chotirmall S.H., Dickson R.P., Reyes L.F. (2023). Exploring the complex relationship between the lung microbiome and ventilator-associated pneumonia. Expert Rev. Respir. Med..

[38] van der Ploeg G.R., Brandt B.W., Keijser B.J.F., van der Veen M.H., Volgenant C.M.C., Zaura E., Smilde A.K., Westerhuis J.A., Heintz-Buschart A. (2024). Multi-way modelling of oral microbial dynamics and host-microbiome interactions during induced gingivitis. npj Biofilms Microbiomes.

[39] Yang X., Zhang S., Ning T., Wu J. (2025). *Fusobacterium nucleatum* in Health and Disease. MedComm (2020).

[40] Leonardi I., Gao I.H., Lin W.Y., Allen M., Li X.V., Fiers W.D., De Celie M.B., Putzel G.G., Yantiss R.K., Johncilla M. (2022). Mucosal fungi promote gut barrier function and social behavior via Type 17 immunity. Cell.

[41] Underhill D.M., Iliev I.D. (2014). The mycobiota: Interactions between commensal fungi and the host immune system. Nat. Rev. Immunol..

[42] Boutin R.C., Petersen C., Woodward S.E., Serapio-Palacios A., Bozorgmehr T., Loo R., Chalanuchpong A., Cirstea M., Lo B., Huus K.E. (2021). Bacterial–fungal interactions in the neonatal gut influence asthma outcomes later in life. eLife.

[43] Rodríguez N., Whitfield-Cargile C.M., Chamoun-Emanuelli A.M., Hildreth E., Jordan W., Coleman M.C. (2021). Nasopharyngeal bacterial and fungal microbiota in normal horses and horses with nasopharyngeal cicatrix syndrome. J. Vet. Intern. Med..

[44] Arastehfar A., Carvalho A., Houbraken J., Lombardi L., Garcia-Rubio R., Jenks J.D., Rivero-Menendez O., Aljohani R., Jacobsen I.D., Berman J. (2021). *Aspergillus fumigatus* and aspergillosis: From basics to clinics. Stud. Mycol..

[45] Rozaliyani A., Antariksa B., Nurwidya F., Zaini J., Setianingrum F., Hasan F., Nugrahapraja H., Yusva H., Wibowo H., Bowolaksono A. (2023). The Fungal and Bacterial Interface in the Respiratory Mycobiome with a Focus on *Aspergillus* spp. Life.

[46] Kanj A.N., Guiance I.R., Kottom T.J., Schaefbauer K.J., Choudhury M., Limper A.H., Skalski J.H. (2024). The commensal fungus *Wallemia mellicola* enhances asthma in mice through Dectin-2. Med. Mycol..

[47] Kanj A., Kottom T., Schaefbauer K., Limper A.H., Skalski J. (2021). Mechanisms by Which Gut-Lung Axis Interactions Enhance Asthma Severity: The Dysbiosis-Associated Fungus *Wallemia mellicola* Signals via Dectin-2/CARD9 Pathway. Am. J. Respir. Crit. CARE Med..

[48] Shi Y., Li Y., Li H., Haerheng A., Marcelino V.R., Lu M., Lemey P., Tang J., Bi Y., Pettersson J.H. (2025). Extensive cross-species transmission of pathogens and antibiotic resistance genes in mammals neglected by public health surveillance. Cell.

[49] Jia W., Xie G., Jia W. (2018). Bile acid-microbiota crosstalk in gastrointestinal inflammation and carcinogenesis. Nat. Rev. Gastroenterol. Hepatol..

[50] Thibaut M.M., Bindels L.B. (2022). Crosstalk between bile acid-activated receptors and microbiome in entero-hepatic inflammation. Trends Mol. Med..

[51] Sanlier N., Yildiz E., Ozler E. (2024). An Overview on the Effects of Some Carotenoids on Health: Lutein and Zeaxanthin. Curr. Nutr. Rep..

[52] Alireza M., Marzieh B., Sepideh S., Azam B. (2017). Carotenoids: Biochemistry, pharmacology and treatment. Br. J. Pharmacol..

[53] Yu W., Wang K., He Y., Shang Y., Hu X., Deng X., Zhao L., Ma X., Mu X., Li R. (2024). The potential role of lung microbiota and lauroylcarnitine in T-cell activation associated with checkpoint inhibitor pneumonitis. EBioMedicine.

[54] Li J., Huang F., Zhou Y., Huang T., Tong X., Zhang M., Chen J., Zhang Z., Du H., Liu Z. (2024). Comprehensive lung microbial gene and genome catalogs assist the mechanism survey of *Mesomycoplasma hyopneumoniae* strains causing pig lung lesions. iMeta.

[55] Elizabeth A.A. (2022). Bacterial–fungal interactions: Bacteria take up residence in the house that Fungi built. Curr. Biol..

[56] Lapiere A., Richard M.L. (2022). Bacterial-fungal metabolic interactions within the microbiota and their potential relevance in human health and disease: A short review. Gut Microbes.

[57] Nogueira F., Sharghi S., Kuchler K., Lion T. (2019). Pathogenetic Impact of Bacterial–Fungal Interactions. Microorganisms.

[58] Marios A., Eleftherios M. (2015). Fungal-bacterial interactions and their relevance in health. Cell. Microbiol..

[59] Leclair L.W., Hogan D.A. (2010). Mixed bacterial-fungal infections in the CF respiratory tract. Med. Mycol..

[60] Peters B.M., Noverr M.C. (2013). *Candida albicans*-*Staphylococcus aureus* polymicrobial peritonitis modulates host innate immunity. Infect. Immun..

[61] Huang S., Wang H., Tian J., Qin M., Gao R., Zhao B., Wang J., Wu H., Xu H. (2025). The Impact of Bacterial–Fungal Interactions on Childhood Caries Pathogenesis. Pathogens.

