# Supplementary material for: Nasopharyngeal Bacterial–Fungal Dysbiosis in Respiratory-Diseased Endangered Forest Musk Deer (Moschus berezovskii)
Source: Biology (Basel). 2026 Apr 6;15(7):587. doi: 10.3390/biology15070587 (PMC13072165; doi:10.3390/biology15070587)
Supplement: Supplementary file 1 [file biology-15-00587-s001.zip › Figure S1.pdf]

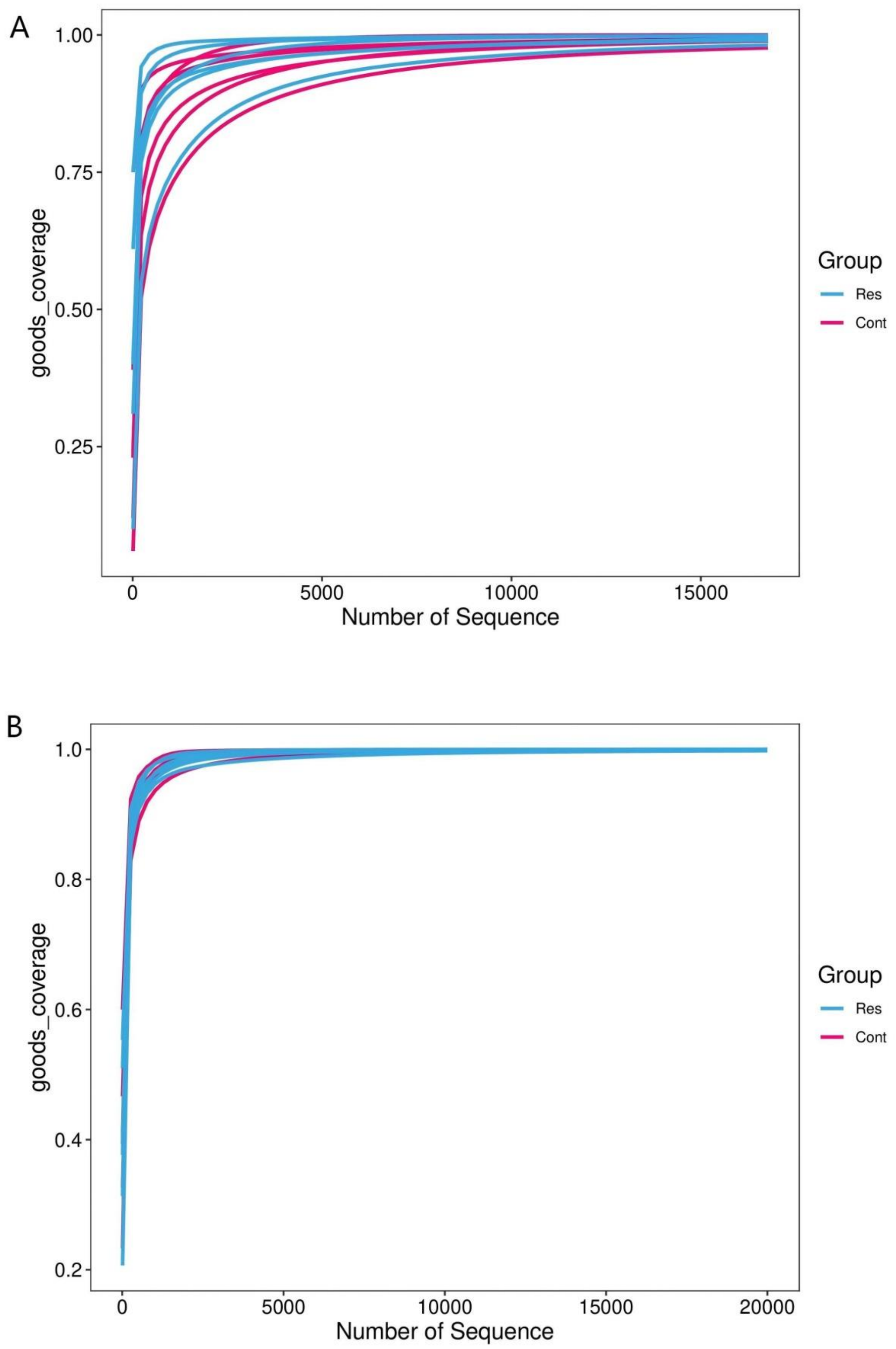

Supplementary Figure S1 Rarefaction curve of 16S rRNA gene sequencing.

A:Rarefaction curve of 16S rRNA gene sequencing.

B: Coverage curve of ITS sequence sequencing
